# Neoadjuvant chemotherapy with modified FOLFOXIRI for locally advanced rectal cancer to transform effectively EMVI and MRF from positive to negative: results of a long-term single center phase 2 clinical trial

**DOI:** 10.1186/s12885-023-11103-x

**Published:** 2023-06-27

**Authors:** Wen Zhang, Haitao Zhou, Jun Jiang, Yuelu Zhu, Shuangmei Zou, Liming Jiang, Yuan Tang, Jianwei Liang, Yongkun Sun, Zhichao Jiang, Wang Qu, Ying Li, Aiping Zhou

**Affiliations:** 1 Department of Medical Oncology, National Cancer Center/National Clinical Research Center for Cancer/Cancer Hospital, Chinese Academy of Medical Sciences and Peking Union Medical College, No.17, Nanli, Panjiayuan, Chaoyang District, Beijing, 100021 China; 2 Department of Colorectal Surgery, National Cancer Center/National Clinical Research Center for Cancer/Cancer Hospital, Chinese Academy of Medical Sciences and Peking Union Medical College, Beijing, 100021 China; 3 Department of Diagnostic Radiology, National Cancer Center/National Clinical Research Center for Cancer/Cancer Hospital, Chinese Academy of Medical Sciences and Peking Union Medical College, Beijing, 100021 China; 4 Department of Pathology, National Cancer Center/National Clinical Research Center for Cancer/Cancer Hospital, Chinese Academy of Medical Sciences and Peking Union Medical College, Beijing, 100021 China; 5 Department of Radiotherapy, National Cancer Center/National Clinical Research Center for Cancer/Cancer Hospital, Chinese Academy of Medical Sciences and Peking Union Medical College, Beijing, 100021 China

**Keywords:** Neoadjuvant chemotherapy, Locally advanced rectal cancer, Pathological complete response, FOLFOXIRI, Down-staging

## Abstract

**Purpose:**

Chemoradiotherapy (CRT) remains the standard treatment for locally advanced rectal cancer (LARC). This phase 2 clinical trial was designed to evaluate the efficacy and safety of neoadjuvant triplet chemotherapy with mFOLFOXIRI (folinic acid, 5-fluorouracil, oxaliplatin, and irinotecan) in LARC.

**Patients and methods:**

The patients with LARC (the lower edge more than 5 cm from the anal verge) received up to 5 cycles of mFOLFOXIRI. MRI was performed to assess the baseline and postchemotherapy TN stage. Radical resection was performed within 4–6 weeks from the last dose of chemotherapy if the tumor shrank or remained stable. Adjuvant chemotherapy with mFOLFOX6 or XELOX was recommended. Postoperative radiation was planned for R1 resection, ypT4b, ypN2 and a positive CRM. The primary endpoint was the pathological complete response (pCR) rate.

**Results:**

From February 2016 to March 2019, 50 patients were enrolled. Forty-eight (96%) were clinically node-positive, 28 (56.5%) with MRF invasion and 39 (78.4%) were EMVI positive. The median cycle of neoadjuvant mFOLFOXIRI chemotherapy was 5 (range,1–5). A total of 46/50 (92%) patients underwent total mesorectal excision (TME) surgery, all with R0 resection. The pCR rate was 4.3% (2/46). Twenty-three of 46 (50%) patients with cN + achieved a pathological node-negative status. The proportions of pathologically positive CRM and EMVI were 2.2% and 34.7%, respectively. Adjuvant radiotherapy was given to 14/46 (30.4%) patients. The most common Grade 3 or > toxicities included neutrocytopenia (50%), leukopenia (14%) and diarrhea (12%) during the neoadjuvant chemotherapy period. Clinically meaningful postoperative complications included pneumonia (*n* = 1), pelvic infection (*n* = 1) and anastomotic fistula (*n* = 1). With a median follow-up time of 51.2 months, local recurrences and distant metastases were confirmed in 3 (6.5%) and 9 (19.6%) of cases, respectively. The 3-year disease free survival (DFS) and overall survival (OS)rates were 75.8% and 86.8%.

**Conclusion:**

Neoadjuvant chemotherapy with mFOLFOXIRI yielded a significant down-staging effect and seemed to be effective in eliminating EMVI and transforming the positive MRF to negative in LARC. The survival results are promising. The long-term follow-up showed promising DFS and OS rates accompanied by a favorable safety profile.

**Trial registration:**

ClinicalTrials.gov identifier: NCT03443661, 23/02/2018.

**Supplementary Information:**

The online version contains supplementary material available at 10.1186/s12885-023-11103-x.

## Introduction

Over 1.9 million new colorectal cancer cases and 915,880 deaths were estimated to occur worldwide in 2020 [1]. Overall, colorectal cancer ranked third in terms of its incidence and second in terms of mortality [1]. New cases of rectal cancer accounted for 38.9% of all colorectal cancers [1]. In China, the incidence of colorectal cancer was ranked third in 2015 and its incidence is currently increasing [2]. The proportion of rectal cancer patients is significantly higher in China than in Europe and the US, although had declined from 72.6% in the 1980s to 49.6% in 2013 [3, 4].

Fluorouracil-based preoperative chemoradiotherapy (CRT) followed by total mesorectal excision (TME) and adjuvant chemotherapy remains the standard treatment for locally advanced rectal cancer (LARC) [5]. In spite of improvements in local disease control, no decrease in the incidence of distant metastases was observed and the beneficial effects on overall survival (OS) remains unclear [6, 7]. The results of phase 3 clinical trials of total neoadjuvant therapy (TNT) reported more significant tumor regression and a reduced incidence of distant metastases, hence TNT has become another standard treatment option. All these studies had high completion rates of chemotherapy over 95% in common, even when the FOLFIRINOX triple regimen was adopted in the PRODIGE 23 study [8]. Furthermore, TNT provided a significantly greater pathological complete response (pCR) rate compared to CRT, which were 27.8% *vs* 12.1% in the PRODIGE 23 study [8], and 28.4% *vs* 14.3% in the RAPIDO studies, respectively [9]. In addition, a watch and wait strategy may be implemented if clinical complete regression (cCR) was achieved, which makes changes in the surgical procedure and organ preservation possible.

After consideration of the long-term side effects elicited by radiotherapy, several studies have endeavored to explore the role of neoadjuvant chemotherapy (nCT) as monotherapy. In the FOWARC study, though the neoadjuvant with mFOLFOX6 alone achieved a postoperative pCR rate of 6.6%, the rates of R0 resection (89.4% *vs* 90.7%), anal preservation (89.5% *vs* 84.4%), and 3-year local recurrence (8.3% *vs* 8.0%) were similar compared to CRT, as well as producing less postoperative complications [10]. The 2021 ESMO congress CONVERT trial enrolled patients without mesorectal fascia (MRF) invasion based on pelvic magnetic resonance images (MRI) at baseline [11]. Four cycles of XELOX regimen chemotherapy showed comparable pCR (11% *vs* 13.8%) and downstaging rates (40.8% *vs* 45.6%), with capecitabine-based CRT in a neoadjuvant setting. While the occurrences of preventive diverting ileostomy and peri-operative distant metastasis were significantly reduced in nCT compared to CRT patients (52.3% *vs* 63.6%, *P* = 0.008; 0.7% *vs* 3.1%, *P* = 0.034, respectively). It seemed nCT alone was promising according to the results reported (vide supra).

Given the low pCR rate with mFOLFOX6 in the FOWARC study, we conducted a phase 2 clinical trial to evaluate the efficacy and safety of mFOLFOXIRI, a more intensive and more active triplet regimen proven to be effective for the treatment of metastatic colorectal cancer [12, 13]. We also further explored the role of nCT alone as neoadjuvant treatment for patients with LARC from December 2015. Patient enrollment ended in March 2019. Herein, the long-term follow-up results are reported.

## Materials and methods

### Study design

A prospective single-arm phase 2 study and its protocol was registered at ClinicalTrials.gov (ClinicalTrials.gov identifier: NCT03443661,23/02/2018) and approved by the central ethics committee of the Cancer Hospital of Chinese Academy of Medical Science, Beijing, China, with ethical approval number: 15–134/1061.The study was conducted in accordance with the principles of the Declaration of Helsinki and Good Clinical Practice. All patients included in the study provided written informed consent.

### Patient selection

The main inclusion criteria were: treatment naïve and pathologically confirmed rectal adenocarcinoma cases; aged between 18 and 70 years; an Eastern Cooperative Oncology Group performance status of 0 to 1; clinical stage II (T3-4aN0) or III (T1-4a N1-2) determined by contrast-enhanced pelvic MRI; no distant metastases confirmed by contrast-enhanced computed tomography (CT) scans of the chest, abdomen and pelvis; distal edge of tumor between 5 and 15 cm from the anal verge; bone marrow, liver and kidney functions before treatment that met the following criteria: neutrophils ≥ 2 × 10^9^/L; platelets ≥ 100 × 10^9^/L; hemoglobin ≥ 9.0 g/dL; total bilirubin ≤ 1.5 times the upper limit of normal (ULN); ALT ≤ 1.5 ULN; AST ≤ 1.5 ULN; and Cr ≤ 1.0 ULN.

The main exclusion criteria were: clinical staging T4b; intestinal obstruction and perforation; serious bleeding caused by tumor, treatment with pelvic radiotherapy; previous systemic chemotherapy within 5 years; other malignant tumors within 5 years (with the exception of cervical cancer in situ or adequately treated non-melanoma skin cancers).

### Treatment regimens

A modified FOLFOXIRI regimen (irinotecan 150 mg/m^2^, oxaliplatin 85 mg/m^2^, leucovorin 200 mg/m^2^) was administered on day 1, including a fluorouracil 2,400 mg/m^2^ continuous infusion for 48 h started on day 1 and then repeated every 14 days. The patients were given up to 5 cycles of nCT if the tumor shrank or remained stable at the initial evaluation after completion of the first 3 cycles. Pelvic MRI was evaluated at baseline and after nCT by an independent imaging expert group to determine the response. Patients who had any clinical evidence of progression during chemotherapy or who were intolerant to chemotherapy were submitted to the multidisciplinary team (MDT) discussion for further management (for example, to receive CRT or surgical resection).

Radical resection was performed within 4–6 weeks after nCT. The mFOLFOX6 (oxaliplatin 85 mg/m^2^, leucovorin 200 mg/m^2^ followed by fluorouracil 400 mg/m^2^ and fluorouracil 2,400 mg/m^2^ administered intravenously every 48 h for 14 days) up to 7 cycles or XELOX (oxaliplatin 130 mg/m^2^ administered intravenously on day 1, oral capecitabine 1,000 mg/m^2^ on days 1 to 14 every 21 days) for up to 4 cycles was administered as adjuvant chemotherapy for patients with R0 resection within 4–6 weeks after resection. Postoperative CRT was given to patients with pT4b, pN2, CRM ≤ 1 mm, R1 or R2 resection. MDT panelists determined whether CRT should be administered to patients with pT3N0 or pT1-3N1, based on tumor location, the baseline clinical stage or high-risk factors.

### Evaluation of the study and its end points

The primary endpoint was the pCR rate, which was defined as the absence of tumor cells in both primary and local lymph nodes. The extent of residual tumor in the resected TME specimens were classified according to the TNM staging system of the American Joint Committee on Cancer version 7. In addition to TN staging, the pathological examination report also included the status of extramural venous invasion (EMVI) and CRM. Tumor regression was assessed by the DWORAK grading system [14]. The pathological evaluation was confirmed by two experienced pathologists (Doctor SM, Z and YL, Z).

The secondary endpoints included the R0 resection and local recurrence rates, disease free survival (DFS), OS, safety of nCT and postoperative complications. Safety was evaluated by investigation according to the NCI CTC AE 4.0 guidelines. Dose reduction was allowed in cases of severe toxicity which was mainly manifested by grade 4 neutropenia or grade 3 febrile neutropenia, diarrhea, or mucositis. Prophylaxis with G-CSF is not recommended unless grade 4 neutropenia with febrile neutropenia occurred during the previous cycle of treatment. The incidence of complications within 1 month after surgery was recorded and evaluated according to the Clavien-Dindo grading system.

### Statistical considerations

This was a single-arm study. According to the FOWARC study, nCT with mFOLFOX6 treatment alone led to a 6.6% pCR rate. We hypothesized that mFOLFOXIRI would further improve the rate of pCR to 16%. With a 1-sided type I error of 0.05 and a power of 80%, and with a 10% dropout rate considered, the intended number of patients was 46. All statistical analyses were conducted using SPSS ver. 22.0 software.

## Results

### Baseline characteristics

A total of 50 patients were recruited between February 2016 and March 2019 who had a median age of 57 years (range, 26–66); 18 were female (36%). There were 19 patients with upper rectal cancer (38%) and 31 with middle rectal cancer (62%). All were clinical T3 and T4a. There were 48 (96%) clinical node-positive patients and 25 (50%) clinical stage N2 patients. Forty-eight patients were at clinical stage III (96%), except for 2 stage II patients (4%) (Table 1).

**Table 1:** Baseline demographics and clinical characteristics (*N* = 50)

Characteristic	N (%)
Age, years	
Median (range)	57 (26–66)
Sex	
Male	32 (64)
ECOG PS	
0	36 (72)
1	14 (28)
Clinical T category	
T3a	0
T3b	14 (28)
T3c	15 (30)
T3d	1 (2)
T4a	20 (40)
Clinical N category	
N0	2 (4)
N1	23 (46)
N2	25 (50)
cTNM Staging	
II	2 (4)
III	48 (96)
Distance from the anal verge, cm	
10–15 cm	19 (38)
5–10 cm	31 (62)

### Treatment administration

#### Neoadjuvant chemotherapy

**Fig. 1:**
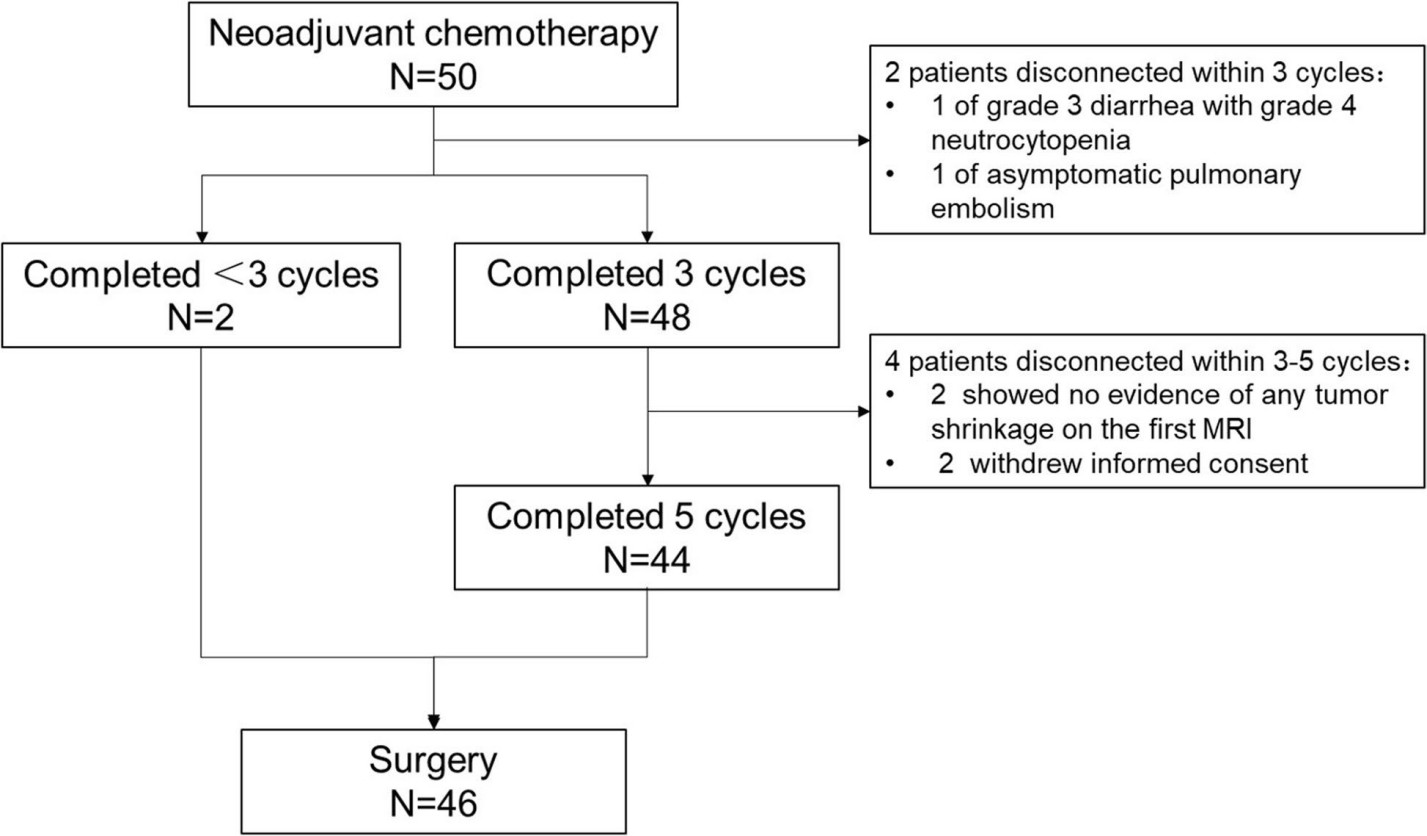
Patients flow diagram

The median number of nCT cycles was 5 (range: 1–5). Six of 50 (12%) patients failed to complete 5 cycles of nCT. Two patients discontinued because of adverse events (1 case of grade 3 diarrhea with grade 4 neutropenia related to nCT and 1 asymptomatic pulmonary embolism unrelated to the treatment). Two patients showed no evidence of any tumor shrinkage on the first evaluation with MRI, and then received CRT and 2 patients withdrew informed consent. The patients flow diagram is shown in Fig. 1.

#### Surgery and adjuvant treatment

The number of patients who underwent surgery was 46 of 50 (92%), including 2 patients treated for < 3 drug cycles because of adverse events (1 patient underwent surgery after 2 cycles, and 1 patient underwent surgery after 1 cycle of mFOLFOXIRI chemotherapy and 1 cycle of XELOX). The completion rate of adjuvant chemotherapy was 91.3%, since 4 of 46 patients who underwent surgery did not receive therapy. Only 1 patient was given capecitabine alone for adjuvant chemotherapy after recovery from an anastomotic fistula, while all the other patients accepted the XELOX adjuvant chemotherapy. The median time to start adjuvant chemotherapy is 35 (range 30–60) days after surgery. Adjuvant radiotherapy was given to 14/46 (30.4%) patients according to the opinion of MDT, all of whom presented with a distal edge of tumor < 12 cm, including 5 pN2, 1 CRM-positive, 7 pN1 and 2 pN0 patients.

### Efficiency and safety results

All the 46 patients received R0 resection. The pCR rate was 4.3% (2/46). A summary of the pathological results is presented in Table 2. Adverse events related to the nCT (incidence rate ≥ 10%) were shown in Table 3. The most common Grade 3 or more toxicities included neutropenia (50%), leukopenia (14%), and diarrhea (12%). Overall, 7/50 (14%) of patients required a dose reduction due to treatment-related adverse events. During neoadjuvant chemotherapy, no patient received prophylactic G-CSF. G-CSF was used in 10 patients (10/50, 20%) due to grade 3 or 4 neutropenia in neoadjuvant setting. Clinically meaningful postoperative complications included pneumonia (*n* = 1), pelvic infection (*n* = 1), and anastomotic fistula (*n* = 1). According to the Clavien-Dindo classification, the complications were all Grade 1 (6.5%).

**Table 2:** Summary of study outcomes (*N* = 46)

Variables	N (%)
pCR	2 (4.3)
R0 resection	46 (100)
Tumor downstaging (to Stage 0/I/II)	23 (50)
Dworak TRG	
4	2 (4.3)
3	5 (10.9)
2	17 (36.9)
1	22 (47.8)
0	0
Post-operative complications^a^	3 (6.6)
Anastomotic fistula	1 (2.2)
Pneumonia	1 (2.2)
Pelvic infection	1 (2.2)

^a^According to the Clavien-Dindo classification, the complications were all grade I

**Table 3:** Summary of adverse events (*N* = 50)

Events	Any Grade N (%)	Grade 3/4 N (%)
Neutropenia	38 (76)	15 (50)
Leukopenia	30 (60)	7 (14)
Diarrhea	26 (52)	6 (12)
Nausea	25 (50)	1 (2)
Elevated ALT	18 (36)	2 (4)
Peripheral neurotoxicity	15 (30)	0
Anemia	14 (28)	0
Loss of appetite	14 (28)	0
Vomiting	11 (22)	1 (2)
Fatigue	10 (20)	0
Thrombocytopenia	7 (14)	2 (4)
Elevated bilirubin	2 (4)	0

### Survival

**Fig. 2:**
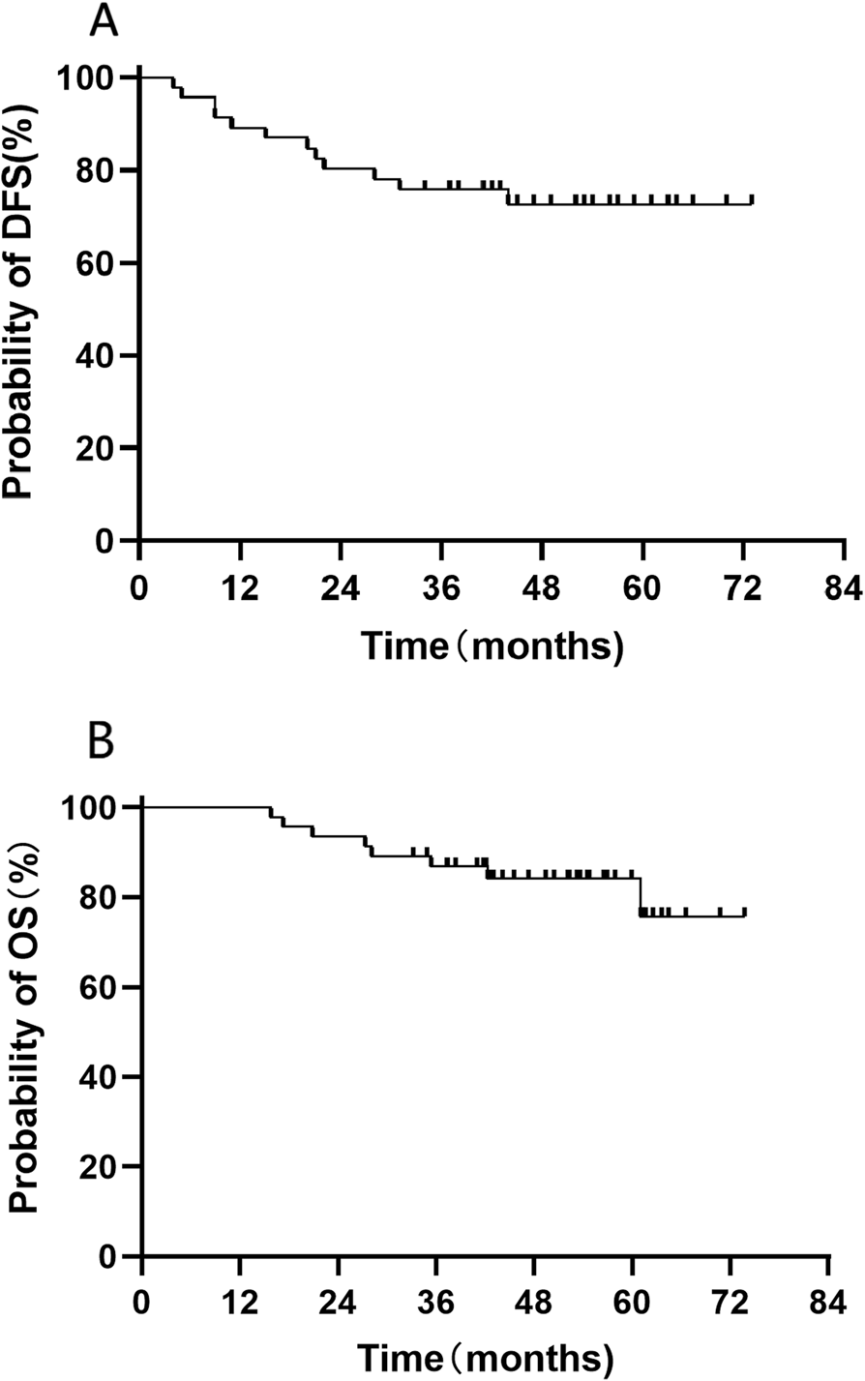
Kaplan–Meier estimate of (**A**) disease-free survival and (**B**) overall survival

As of January 31, 2022, the median follow-up time was 51.2 months for the 46 patients who received R0 resection, with local recurrences confirmed in 3 (6.5%) patients. Nine of 46 (19.6%) patients developed metastatic disease, including 6 with pulmonary metastases, 1 liver metastasis and 2 retroperitoneal lymph node metastases. Until the last follow-ups, the median DFS and OS had not been reached while the 3-year DFS and OS rates were 75.8% and 86.8%, respectively (Fig. 2).

The status of MRF and EMVI on MRI at baseline and after nCT were evaluated retrospectively. These results were compared with pathological status after surgery (Table 4). Survival analysis was also performed based on EMVI, MRF and lymph node status.

**Table 4:** Characteristics evaluated by MRI at baseline and after neoadjuvant chemotherapy and the pathological characteristics (*N* = 46)

Characteristics	Baseline by MRI, N (%)	After neoadjuvant chemotherapy by MRI, N (%)	Pathology, N (%)
T Stage			
T0	0 (0)	0 (0)	2 (4.3)
T1	0 (0)	0 (0)	0 (0)
T2	0 (0)	6 (13)	4 (8.7)
T3	27 (34.7)	37 (80.4)	35 (76.1)
T4	19 (65.3)	3 (6.5)	5 (10.9)
N Stage			
N0	2 (4.3)	22 (47.8)	23 (50)
N1	22 (47.8)	20 (43.4)	12 (26.1)
N2	22 (47.8)	4 (8.7)	11 (23.9)
TNM Stage			
0	0	0	2 (4.3)
I	0	6 (13)	3 (6.5)
II	2 (4.3)	16 (34.8)	18 (39.1)
III	44 (95.7)	24 (52.2)	23 (50)
EMVI			
Positive	36 (78.4)	15 (32.6)	16 (34.8)
Negative	10 (21.6)	31 (67.4)	30 (65.2)
MRF/CRM			
Positive	26 (56.5)	4 (8.7)	1 (2.2)
Negative	20 (43.5)	42 (91.3)	45 (97.8)

### EMVI, MRF and lymph nodes with survival

**Fig. 3:**
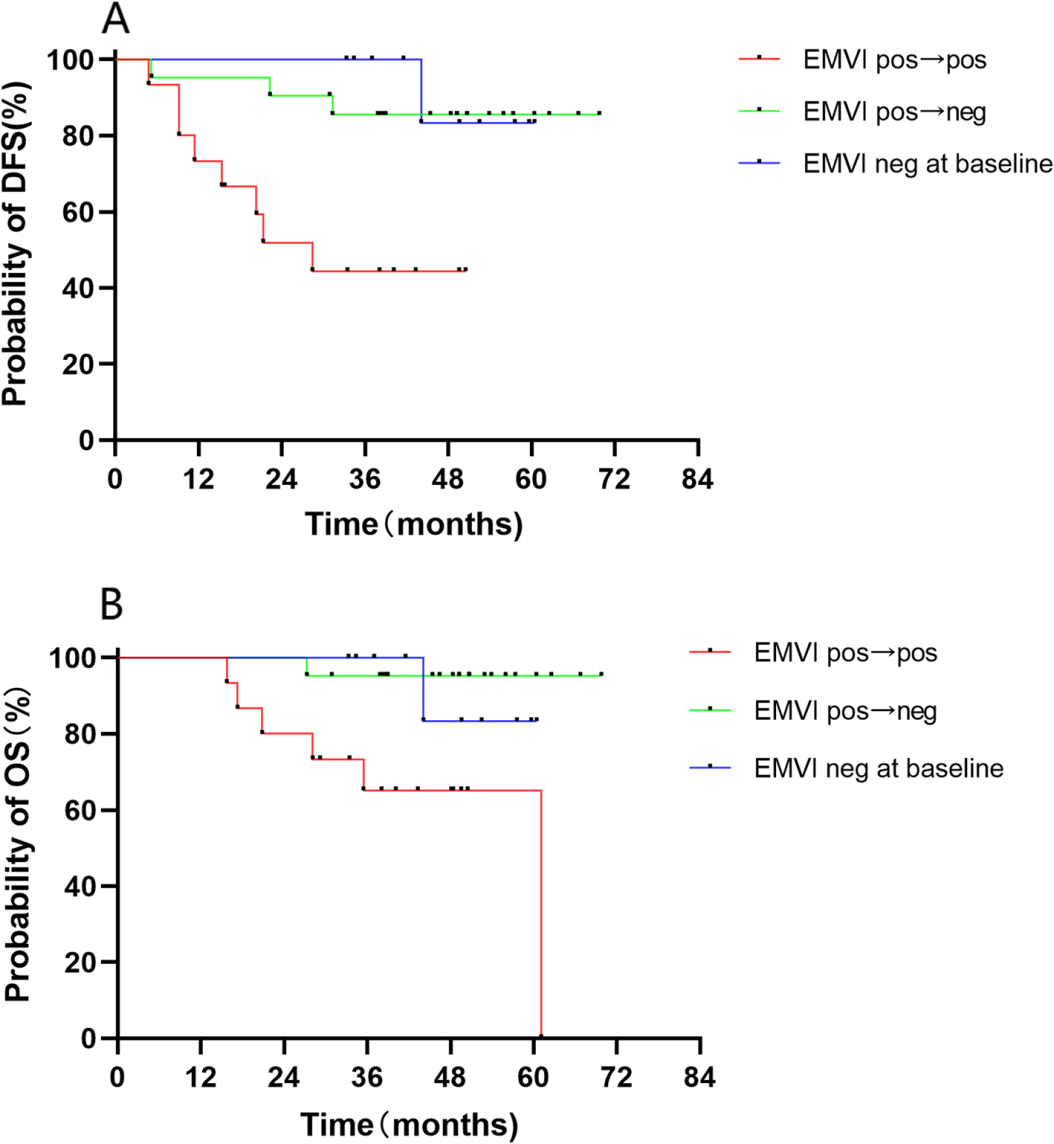
Kaplan–Meier estimate for EMVI status with (**A**) disease-free survival and (**B**) overall survival

The incidence of positive EMVI revealed by MRI decreased from 78.4% at baseline to 32.6% after nCT and was 34.8% on pathology evaluations. Patients with EMVI status transforming from positive at baseline to negative after nCT (*n* = 21) had remarkably longer DFS (*P* = 0.006) and OS (*P* = 0.001) compared to those with EMVI positive all along (*n* = 15), and also had similar DFS *(P* = 0.710) and OS (*P* = 0.490) compared to the patients with EMVI negative at baseline (*n* = 10) (Fig. 3).

**Fig. 4:**
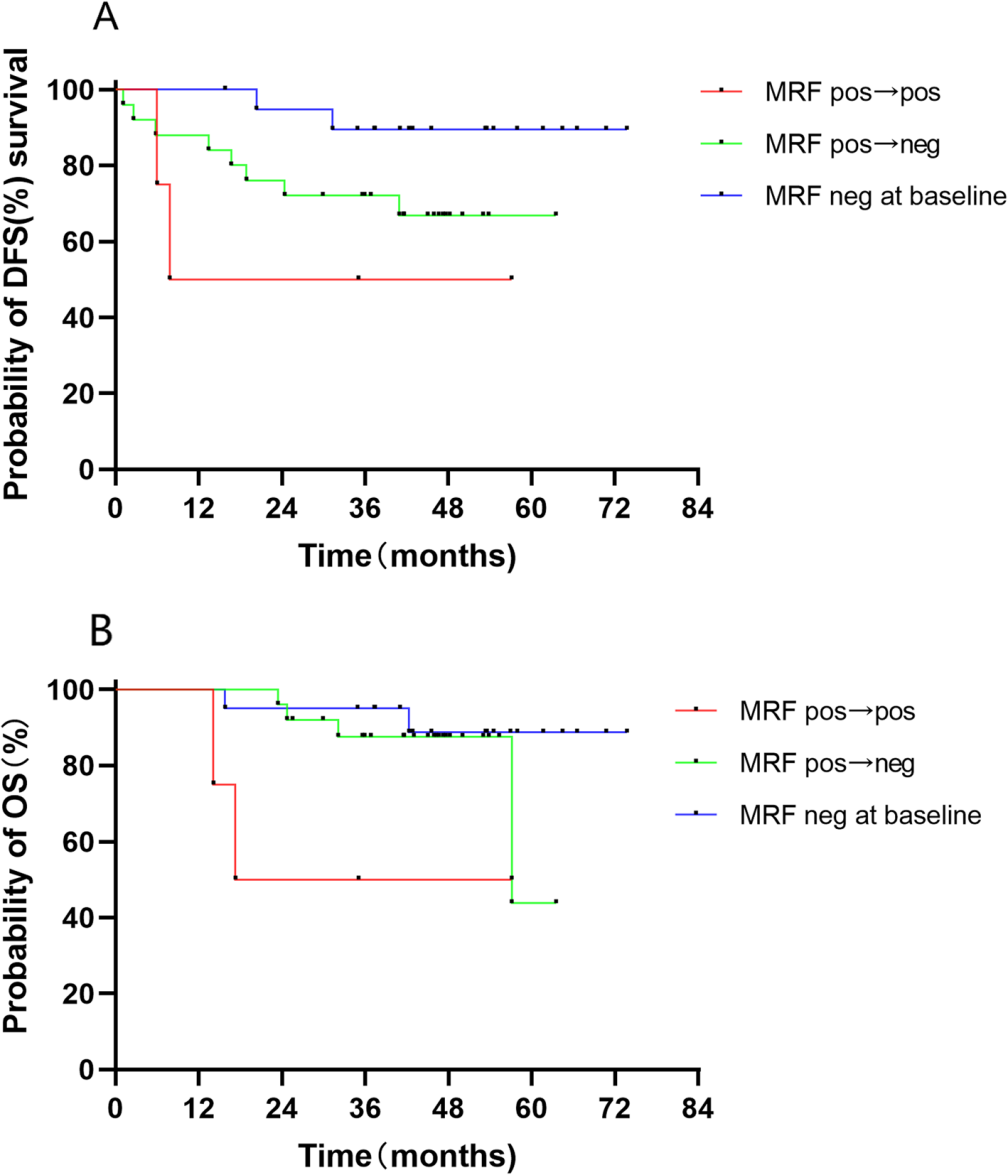
Kaplan–Meier estimate for MRF status with (**A**) disease-free survival and (**B**) overall survival

The incidence of positive MRF detected by MRI decreased from 56.5% at baseline to 8.7% after nCT and the incidence of positive CRM was 2.2% on pathology evaluations. Patients with MRF status transforming from positive at baseline to negative after nCT (*n* = 22) had numerically longer DFS and OS compared to those who were always MRF positive (*n* = 4), but the sample size was too small to perform a rigorous statistical analysis. Similar DFS (*P* = 0.34) and OS (*P* = 0.42) were achieved compared to those who were MRF negative at baseline (*n* = 20) (Fig. 4).

**Fig. 5:**
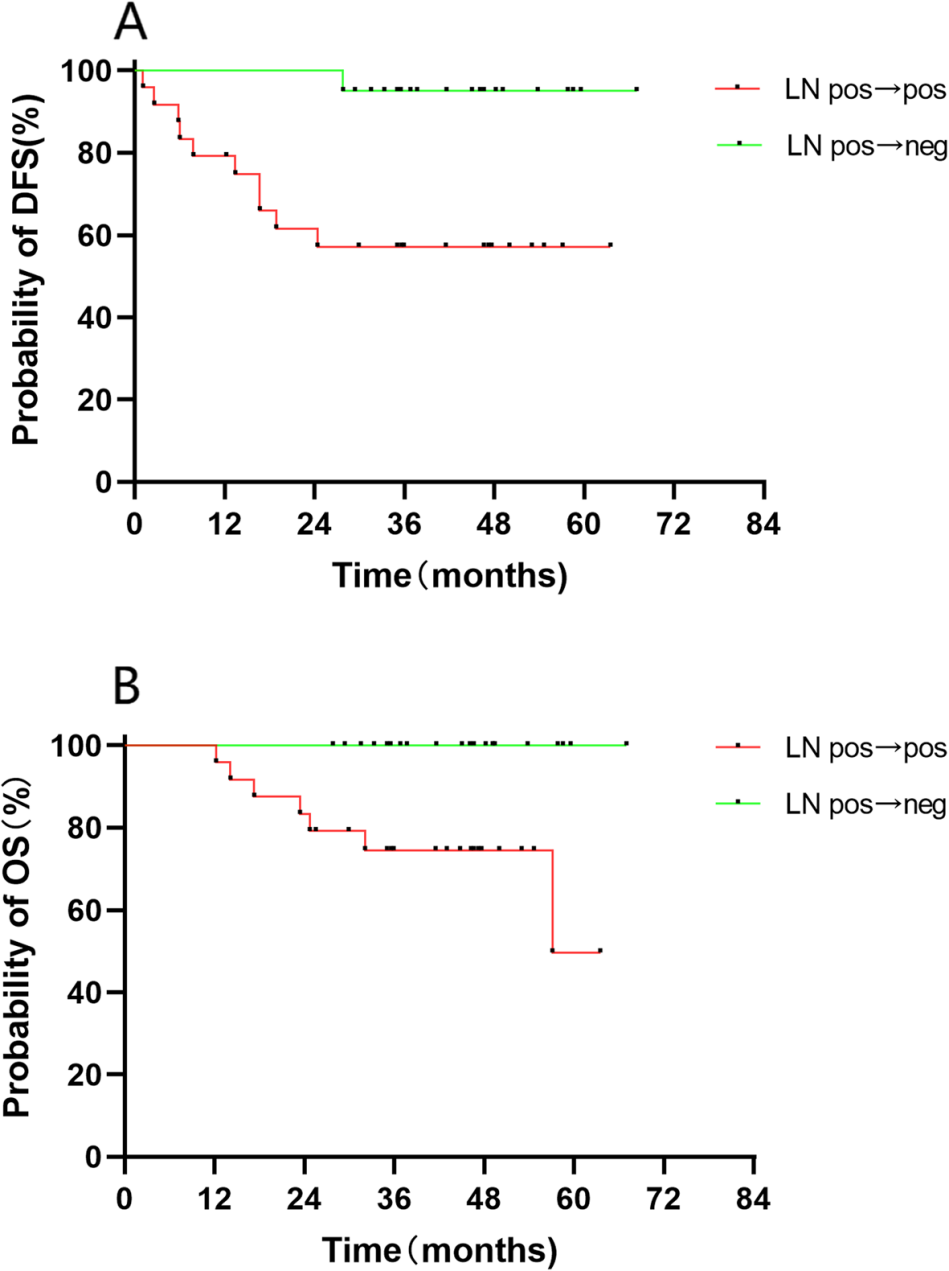
Kaplan–Meier estimate for lymph node (LN) status with (**A**) disease-free survival and (**B**) overall survival

The incidence of MRI positive nodes decreased from 95.6% at baseline to 52.1% after nCT, and the incidence of positive nodes was 50% on pathology evaluations. Patients with lymph nodes status transforming from positive at baseline to negative after nCT (*n* = 20) had remarkably longer DFS (*P* = 0.004) and OS (*P* = 0.005) times compared to those patients who were always lymph node positive (*n* = 24) (Fig. 5). There were only 2 patients with negative lymph nodes at baseline and therefore they were not evaluated.

## Discussion

In the present study, nCT with mFOLFOXIRI therapy for patients with LARC achieved a pCR rate of 4.3%, which did not meet the primary endpoint. The pCR rate was similar to the 6.6% for mFOLFOX6 in the FOWARC study and was lower than the 17.4% of mFOLFOXIRI in the FORTUNE study [15], which was also carried out by the FOWARC team. The low pCR rate in our study may be partly related to the higher proportion of patients with high-risk factors and a heavy tumor burden. Indeed, the baseline characteristics in our study showed 96% node-positive and 50% cN2. Retrospective evaluation found that the positive rates of EMVI and MRF were as high as 78.4% and 56.5%, respectively. However, the baseline data from the FOWARC study included 72.1% node-positive, 26% cN2 and 31.4% MRF invasion patients, all of which were lower than in our study. The baseline characteristics of the FORTUNE study showed 84% node-positive, 45.7% cN2 and 29.2% MRF invasion, while EMVI data were not reported. In addition, there has been no commonly recognized optimal dosage of FOLFOXIRI to date. A phase 1 dose-escalation study in Japan reported maximum tolerated dosages as 85 mg/m^2^ for oxaliplatin, 150 mg/m^2^ for irinotecan and 2,400 mg/m^2^ for 5-fluorouracil [16]. We therefore adopted the same dosage regimens in our study. However, the dosage of irinotecan and 5-fluorouracil were both lower than administered in the FORTUNE study (i.e., 150–165 mg/m^2^ irinotecan and 2,800 mg/m^2^ 5-fluorouracil, respectively), which may be one reason for the lower pCR rate observed in our study.

Neoadjuvant radiotherapy is an effective way to improve local control of LARC. In randomized studies of neoadjuvant CRT versus preoperative radiotherapy alone (FFCD9203 [17] and EORTC22921 [18] studies), the pCR rate of neoadjuvant CRT reached 11.4–14% and the 5-year local recurrence rate was 7.6–8.1%, both of which were significantly superior to preoperative radiotherapy alone. Furthermore, in studies related to TNT strategies (PRODIGE 23 and RAPIDO studies), the pCR rate reached 27.8–28%, and the local recurrence rate was 4.8–8.7%. Though the pCR rate was not as high as expected, our results indicated that nCT with 3 to 5 cycles of mFOLFOXIRI resulted in significant downstaging. Among the 44 patients with node-positive tumor at baseline, 20 were node-negative after completion of nCT based on MRI evaluations. In total, 52.1% of the patients were evaluated as node-positive by MRI after nCT, findings consistent with the pathological negative rate of 50% after surgery. Notably, our results also indicated that nCT with mFOLFOXIRI appeared to be quite effective in eradicating EMVI and transforming patients to a negative MRF. MRF invasion and EMVI-positive are among the top high-risk factors for local recurrence and metastasis in rectal cancer [19, 20]. In the present study, the incidence of MRF invasion revealed by MRI showed a sharp decrease from 56.5% at baseline to 8.7% after nCT, and was confirmed to be 2.2% by pathology evaluations. The incidence of positive EMVI also decreased from 78.4% at baseline to 32.6% after nCT and was confirmed to be 34.8% after pathological assessments. It was further found that patients with lymph node and EMVI status transforming from positive at baseline to negative after nCT achieved significantly longer DFS and OS times compared to those with positive status from the outset of the study. MRF status transforming from positive at baseline to negative after nCT had a trend to prolong DFS and OS times. Moreover, nCT with a triplet regimen transformed high-risk patients into low-risk patients, and achieved comparable survival times to that of low-risk patients at baseline. The importance of identifying persistent EMVI at restaging following neoadjuvant CRT or nCT is not entirely understood, however, it has been reported that patients with EMVI positive tumors at baseline that regress to become EMVI negative at restaging have similar survival outcomes and recurrence rates as patients who were EMVI negative at baseline, and that patients who remained EMVI positive at restaging have shorter disease-free survival [21].

On the other hand, re-evaluation of high-risk factors by MRI after nCT may distinguish patients with different prognosis outcomes. GRECCAR-4 was a randomized phase 2 study in which an nCT plan was established based on the degree of tumor shrinkage after intensive induction chemotherapy (FOLFIRINOX) [22]. In the study, 206 patients with LARC received 4 cycles of FOLFIRINOX. The 5-year DFS and OS rate were significantly improved in good responders (*n* = 30) compared to poor responders (*n *= 196). In addition, good responders treated with standard CRT plus surgery had a higher 5-year DFS rate compared to those who received immediate TME (89.5% *vs* 80%), but the 5-year OS rates were comparable (93.3% *vs* 90%). Although GRECCAR-4 study had the limitation of a small sample size, especially arm A (*n* = 16) with good responders without radiotherapy, it revealed that patients with tumor remission and clearance of high-risk factors (such as EMVI) would likely obtain a better prognosis. For such patients, preoperative chemotherapy alone might achieve the same OS as preoperative CRT.

A high completion rate of nCT with mFOLFOXIRI was observed in this study: 96% for 3 cycles and 88% for 5 cycles, with manageable toxicities. The incidence rates of the most concerning grade 3/4 toxicities namely neutropenia and diarrhea were 50% and 12%, respectively. There were no treatment-related deaths. The nCT with mFOLFOXIRI did not bring any additional safety problems to surgery. The incidence rates of anastomotic fistula and postoperative infection were 2.2% and 4.3%, respectively. The rates in this study were similar to those reported in the mFOLFOX alone treatment group in the FOWARC study (7.9% and 7.2%, respectively), but were significantly lower than in the two concurrent chemoradiation groups (19.8% and 16.3%, and 18.1% and 14.8%, respectively).

Nevertheless, 11/46 (23.9%) patients retained ypN2 disease after surgery. Finally, 14 (30.4%) of 46 patients received postoperative radiation with concurrent capecitabine therapy mainly because they had pathologically node-positive (*n* = 12) or CRM-positive (*n* = 1) tumors. With a median follow-up time of 51.2 months, the incidence of local recurrences (6.5%) and distant metastases (19.6%) appeared to be relatively low, which was promising taking into consideration the fact that most of the patients enrolled in this study were at high-risk. The incidence of local recurrences in this study was numerically comparable to those of 5-fluorouracil- or mFOLFOX6-based CRT in the FOAWRC study and the TNT group in the RAPIDO study. The three-year DFS and OS rates were 75.8% and 86.8%, respectively. Likewise, the survival outcomes also appeared promising numerically compared with the results of the FOWARC and RAPIDO studies.

The present research had several limitations. First, it is a phase 2 single-arm study with a limited sample size. Second, a higher proportion of patients with MRF invasion and EMVI-positive were enrolled, which did not reflect real-world incidence. Third, EMVI and MRF status were post analysis rather than prospective research purposes.

In conclusion, nCT with mFOLFOXIRI had a significant down-staging effect on LARC. It was effective in eliminating EMVI, and transforming positive MRF to a negative status, with a favorable safety profile. The long follow-up showed promising DFS and OS times, indicating that initial neoadjuvant treatment with mFOLFOXIRI could allow a certain subset of patients to be free from radiotherapy. This was especially true for those patients bearing high risks who be eliminated after nCT in the re-evaluation and who probably obtaining better survival, and thus, warrant further investigation.

## Supplementary Information

**Additional file 1: Table S1.** Summary of adverse events of adjuvant chemotherapy(*N* = 42*).

## Data Availability

The datasets generated during and analyzed during the current study are not publicly available due to patients ‘ confidentiality but are available from the corresponding author on reasonable request.

## Abbreviations

CRM: Circumferential resection margin
CRT: Chemoradiotherapy
CT: Computed tomography
DFS: Disease free survival
EMVI: Extramural venous invasion
LARC: Locally advanced rectal cancer
MDT: Multidisciplinary team
MRI: Magnetic resonance imaging
nCT: Neoadjuvant chemotherapy
OS: Overall survival
pCR: Pathological complete response rate
TME: Total mesorectal excision
TNT: Total neoadjuvant therapy
ULN: Upper limit of normal
