# Supplementary material for: Neoadjuvant chemotherapy with modified FOLFOXIRI for locally advanced rectal cancer to transform effectively EMVI and MRF from positive to negative: results of a long-term single center phase 2 clinical trial
Source: BMC Cancer. 2023 Jun 27;23:592. doi: 10.1186/s12885-023-11103-x (PMC10294487; doi:10.1186/s12885-023-11103-x)
Supplement: Supplementary file 1 — Additional file 1: Table S1. Summary of adverse events of adjuvant chemotherapy(N = 42*). [file 12885_2023_11103_MOESM1_ESM.docx]

Supplementary file

**Table S1** Summary of adverse events of adjuvant chemotherapy (N = 42*)

| **Events** | **Any Grade**  **N (%)** | **Grade 3/4**  **N (%)** |
| --- | --- | --- |
| Diarrhea | 32 (76) | 3 (7) |
| Nausea | 20 (48) | 1 (2) |
| Hand foot syndrome | 18 (43) | 0 (0) |
| Peripheral neurotoxicity | 16 (38) | 2 (5) |
| Loss of appetite | 8 (19) | 0 |
| Fatigue | 7 (17) | 0 |
| Vomiting | 5 (12) | 0 (0) |
| Leukopenia | 30 (71) | 2(5) |
| Neutropenia | 23 (55) | 0 (0) |
| Elevated ALT | 18 (43) | 0 (0) |
| Anemia | 10 (24) | 0 |
| Thrombocytopenia | 10 (24) | 2 (5) |
| Elevated bilirubin | 5 (12) | 0 |

* 42 patients received ajudant chemotherapy, including 14 patients was given adjuvant radiotherapy. 1 patient was given capecitabine alone, while all the other patients accepted the XELOX adjuvant chemotherapy.
